# Expression of Endoplasmic Reticulum Stress-Related Factors in the Retinas of Diabetic Rats

**DOI:** 10.1155/2012/743780

**Published:** 2011-08-28

**Authors:** Shu Yan, Cui Zheng, Zhi-qi Chen, Rong Liu, Gui-gang Li, Wei-kun Hu, Han Pei, Bin Li

**Affiliations:** Department of Ophthalmology, Tongji Hospital, Tongji Medical College, Huazhong University of Science and Technology, 1095 Jie-fang Road, Wuhan, Hubei Province 430030, China

## Abstract

Recent reports show that ER stress plays an important role in diabetic retinopathy (DR), but ER stress is a complicated process involving a network of signaling pathways and hundreds of factors, What factors involved in DR are not yet understood. We selected 89 ER stress factors from more than 200, A rat diabetes model was established by intraperitoneal injection of streptozotocin (STZ). The expression of 89 ER stress-related factors was found in the retinas of diabetic rats, at both 1- and 3-months after development of diabetes, by quantitative real-time polymerase chain reaction arrays. There were significant changes in expression levels of 13 and 12 ER stress-related factors in the diabetic rat retinas in the first and third month after the development of diabetes, Based on the array results, homocysteine- inducible, endoplasmic reticulum stress-inducible, ubiquitin-like domain member 1(HERP), and synoviolin(HRD1) were studied further by immunofluorescence and Western blot. Immunofluorescence and Western blot analyses showed that the expression of HERP was reduced in the retinas of diabetic rats in first and third month. The expression of Hrd1 did not change significantly in the retinas of diabetic rats in the first month but was reduced in the third month.

## 1. Introduction

Diabetic retinopathy (DR) is one of the severe complications of diabetes leading to loss of vision. Although the pathogenic mechanism of DR has been investigated for many years and a number of theories have been proposed [1, 2], the mechanism of DR remains unknown and needs further exploration. 

Some diabetic patients are susceptible to DR, while others are quite resistant or develop minimal pathological changes [3]. It may be supposed that such DR-resistant patients are protected genetically. The existence of a DR-resistant gene was proposed, and a comparative study was performed of the gene expression between susceptible and resistant DR patients [4]. It was found that many endoplasmic reticulum (ER) stress-related factors are highly expressed in non-DR diabetic patients. 

In our earlier work, we found that P58^IPK^/DNAJC3, an ER stress-related factor, binds to the ER transmembrane protein PERK (protein kinase RNA-activated- (PKR-) like ER kinase), which is normally activated by the ER stress/unfolded protein response. By binding to PERK, P58^IPK^ thereby inhibits its phosphorylation of the *α*-subunit of eukaryotic translation initiation factor 2 (eIF-2*α*) and thus compromises eIF2/EIF2S3's mediator role in the translation of mRNA [5]. In this way, P58^IPK^ inhibits ER stress in the endothelial cells of human retinal vessels. P58^IPK^ also downregulates the expression of vascular endothelial growth factor (VEGF), which is associated with regulation of the pathology of DR [6]. VEGF plays a key role in DR [7, 8] and is regulated at the transcriptional level by the unfolded protein response pathway [9]. Recent reports also show that ER stress plays an important role in DR [10, 11]. Li et al. [12] demonstrated that multiple ER stress markers, including 78 kDa glucose-regulated protein (GRP78), phosphoinositol-requiring transmembrane kinase (IRE)1*α*, and phosphor-eIF2*α* were significantly upregulated in the retinas of animal models of type 1 diabetes and oxygen-induced retinopathy. Our recent work suggests that early progression of DR may be mediated by ER stress, but probably does not involve changes in activating transcription factor (ATF)4 or GRP78 [13]. Together, these studies suggest that although ER stress is involved in the development of DR, its specific pathogenesis is not yet understood.

**Figure 1:**
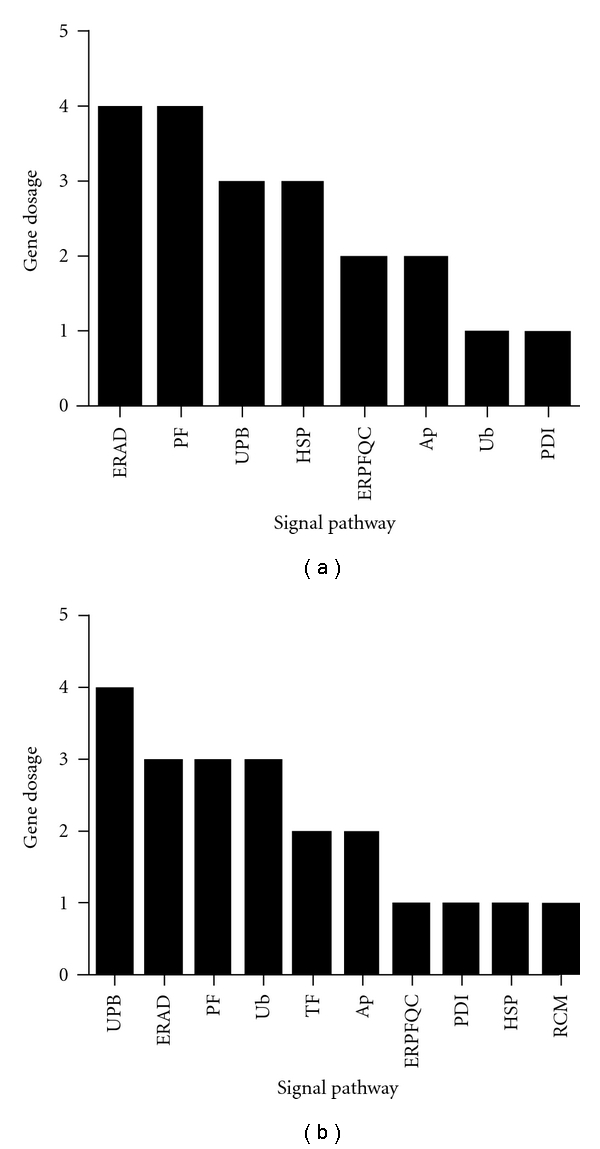
Assessment of the expression of ER stress-related factors in diabetic retinas in the first and third months after the development of diabetes by Q-PCR arrays. (a) the histogram of the expression of different genes in 11 signaling pathways related to ER stress after the first month; (b) the histogram of the expression of different genes in 11 signaling pathways related to ER stress after the third month. Unfolded Protein Binding: UPB, ER Protein Folding Quality Control: ERPFQC, Regulation of Cholesterol Metabolism: RCM, ER-associated degradation: ERAD, Ubiquitination: Ub, Transcription Factors: TF, Protein Folding: PF, Protein Disulfide Isomerization: PDI, Heat Shock Proteins: HSP, Apoptosis: Ap°.

ER stress is a complicated process involving a network of signaling pathways and hundreds of factors that function by triggering the PERK, IRE1 and ATF6 signaling pathways [14–16]. In order to delve into the effects of these ER stress-related factors on DR, we classified them into 11 categories according to function (Figure 1, Table 3), based on Jonikas et al. [17]. We selected 89 ER stress factors from more than 200, based on our work and that of others (Table 4) [13, 17–21]. These factors contain the 11 categories of ER stress. Expression of these factors in the retinas of diabetic rats was determined by quantitative real-time PCR (Q-PCR) arrays to find the specific factors and the ER stress signaling pathways that may play a key role in the pathogenesis of DR.

## 2. Methods

### 2.1. Diabetic Rat Model

Two-month-old male Sprague Dawley rats weighing 150 to 200 g were obtained from the animal center of Huazhong University of Science and Technology. Care, use, and treatment of animals were approved by the laboratory animal center of Huazhong University of Science and Technology. Rats were randomly divided into diabetic and control groups (*n* = 30 per group). The diabetic model was created by intraperitoneal injection of a single dose of streptozotocin (STZ; 65 mg/kg in 0.01 M citrate buffer, pH 4.5) [22]. Nondiabetic rats (the control group) were injected with citrate buffer only. Fasting plasma glucose was examined 3 d after STZ injection, and diabetes was confirmed by a value ≥16.7 mmol/L using Touch Glucometer (Boehringer Mannheim Diagnostics, Indianapolis, IN). Our previous work [13] and that of others [23] have established that in the STZ-induced diabetes model, diabetic retinopathy develops within one month of the development of diabetes. Accordingly, one and three months after the STZ injection, the retinas were separated from the eyes of both the diabetic and control groups. RNA was extracted and assessed using Q-PCR arrays, with 9 rats in each group.

### 2.2. Quantitative Real-Time RNA Polymerase Chain Reaction (Q-PCR) Arrays

The mRNA levels of 96 factors (89 ER stress-related factors and 7 quality control factors) were measured using Q-PCR arrays. Total RNA was extracted from rat retinal tissue using Trizol reagent (Invitrogen, Carlsbad, USA) according to the manufacturer's instructions. RNA was treated with DNAase (Invitrogen, Carlsbad, USA) and purified using Rneasy MinElute Clean-up Kit (Quiagen, Hilden, Germany). The cDNA was then synthesized using a SuperScript III kit (Invitrogen, Carlsbad, USA). Removing the plate seal from the PCR Array (SABioscience, Frederick, USA) and adding the cocktails to the PCR Array, Q-PCR was performed by using the Hot Star polymerase kit (Qiagen, Venlo, The Netherlands) with SYBR Green technology (ABI, Tampa, FL). PCR reaction buffer was added to a 384-well PCR array plate which was then tightly sealed with an optical adhesive cover. The thermocycling program consisted of 95°C for 10 min, then 40 cycles at 95°C for 15 s, and 60°C for one minute, then compared the differential expression of gene between the two groups.

### 2.3. Immunofluorescence

Immunofluorescence was performed on 5 *μ*m frozen sections. Briefly, retinal sections were incubated with a rabbit anti-HERP (Santa Cruz Biotechnology, Santa Cruz, Calif) or anti-Hrd1 (Biosynthesis Biotechnology, Beijing, China) antibody (1 : 200) at 4°C overnight. This was followed by the secondary antibody, fluorescein-conjugated goat antirabbit IgG (Antigene, Wu Han, China), for one hour. The slides were visualized and photographed under a fluorescence microscope (Olympus, Hamburg, Germany).

### 2.4. Western Blot

Total protein was extracted from rat retinal tissue in 300 *μ*L lysis buffer (50 mM Tris pH 7.5, 0.5 M NaCl, 1% NP-40, 1% sodium deoxycholate monohydrate, 2 mM EDTA, and 0.1% SDS). After centrifugation at 1000 ×g for 3 min, protein extracts were diluted with sample buffer (126 mM Tris HCl pH 6.8, containing 20% glycerol, 4% SDS, 0.005% bromophenol blue, and 5% 2-mercaptoethanol) at a 1 : 1 ratio and boiled for 3 minutes. The samples were fractionated according to size on a 12.5% SDS-polyacrylamide gel, transferred to a nitrocellulose membrane (Millipore, Billerica, Mass), and probed with polyclonal anti-HERP (Santa Cruz Biotechnology, Santa Cruz, Calif) or polyclonal anti-Hrd1 (Biosynthesis Biotechnology, Beijing, China) antibodies. A secondary antibody, goat antirabbit IgG (Biosynthesis Biotechnology, Beijing, China) diluted 1 : 1000, was applied, and the chemiluminescent signal was detected. The same membrane was reused to detect *β*-actin (the internal control) by incubating it with mouse antihuman *β*-actin antibody (Gene, Hong Kong, China). Bands observed on the photography films were analyzed by automatic image analysis. The integrated optical density of each protein band was normalized to that of the corresponding *β*-actin band from the same sample.

### 2.5. Rat Retinal Capillary Endothelial Cell (RRCEC) Culture

RRCECs cultured *in vitro* were prepared as previously described [24]. Two-month-old male Sprague Dawley rats weighing 150–200 g (*n* = 60) were obtained from the animal center of Huazhong University of Science and Technology. After anesthesia, the eyes were removed, and the retinas harvested and homogenized by two gentle up-and-down strokes in a 15 mL homogenizer (Dounce; Bellco Glass, Vineland, NJ). The homogenate was filtered through an 88 *μ*m sieve. The retentate was digested in 0.066% collagenase for 45 min at 37°C. The homogenate was centrifuged (1000 ×g for 10 min), and the pellet was resuspended in endothelial basal growth medium (Invitrogen-Gibco, Grand Island, NY), supplemented with 20% fetal bovine serum, 50 U/mL endothelial cell growth factor (Sigma-Aldrich, St. Louis, Mo), and 1% insulin-transferrin-selenium. RRCECs were cultured in fibronectin-coated dishes and incubated at 37°C in a humidified atmosphere containing 5% CO_2_.

Cultured endothelial cells were characterized by evaluating expression of factor VIII antigen (von Willebrand factor) and determining unchanged morphology under culture conditions by light microscopy. The expression of acetyl-LDL (Ac-LDL) receptors in endothelial cells was measured by adding fluorescence-labeled AC-LDL (Biomedical Technologies, Palatine, Il). Only cells from passages 3 to 7 were used in the experiments.

### 2.6. Cell Immunofluorescence

The RRCECs were grown in 24-well plates in human endothelial serum-free material basal growth medium containing 8.3 mM glucose. Upon attaining 80%, confluency cells were treated with medium containing 25 mM glucose for 2 d. Cells were then fixed with 4% formaldehyde for 15 min and permeabilized in 0.1% Triton X-100 for 10 min. Cells were incubated with primary antibody at 4°C overnight followed by secondary antibody for one hour. The slides were visualized and photographed under a fluorescence microscope (Olympus, Hamburg, Germany).

### 2.7. Statistical Analysis

Normally distributed data were compared using Student's independent samples *t*-test or one-way ANOVA where appropriate. When a significant difference was detected between groups, multiple comparisons of means were performed using the Bonferroni procedure, with type-I error rate at a maximum of 0.017 (0.05/3) adjustment. Statistical analyses were performed using Statistical Package for the Social Sciences (SPSS) 15.0 software (SPSS, Chicago, IL). Data were presented as the mean ± standard deviation (SD). A probability (*P*) value <0.05 was considered statistically significant.

## 3. Results

### 3.1. Q-PCR Arrays

We detected 89 ER stress-related genes and found that the mRNA levels of 13 genes in the diabetic rats changed significantly during the first month (Table 1). We found that in the third month the levels of expression of 12 genes were changed significantly in these diabetic rats (Table 2). The changes in the expression levels of genes corresponded to 8 and 10 categories of signal pathways in the first and third months, respectively (Figure 1 and Table 3). The mRNA expressions of* Erdj4* and *HERP* were lower both in the first and third months.

### 3.2. Expression of HERP and HRD1 in the Retinas of Diabetic Rats

**Figure 2:**
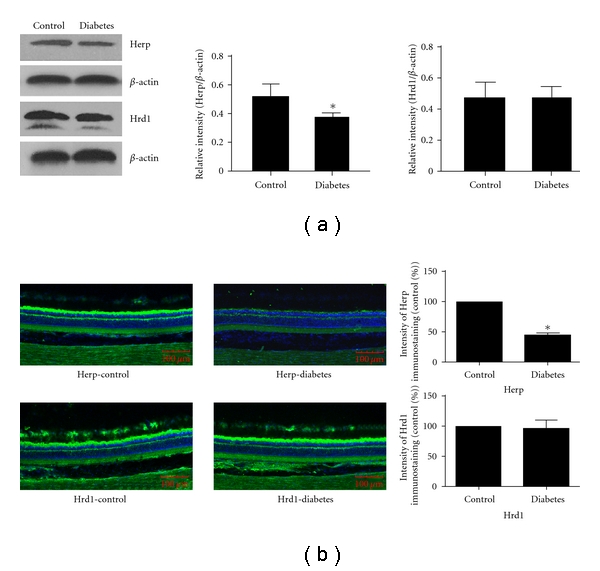
Western blot and immunofluorescence detected the expression of HERP and HRD1 in the first month after the development of diabetes: (a) Western blot detection of the expression of HERP and HRD1 in the first month. The expression of HERP in the diabetic group was less than that of the control group (*P* = 0.004); Hrd1 expression was similar in both groups (*P* = 0.338). (b) Immunofluorescence detection of the expression of HERP and HRD1 in the first month. The expression of HERP in the diabetic group was less than that of the control group (*P* = 0.008) Hrd1 expression was similar in both groups (*P* = 0.572).

**Figure 3:**
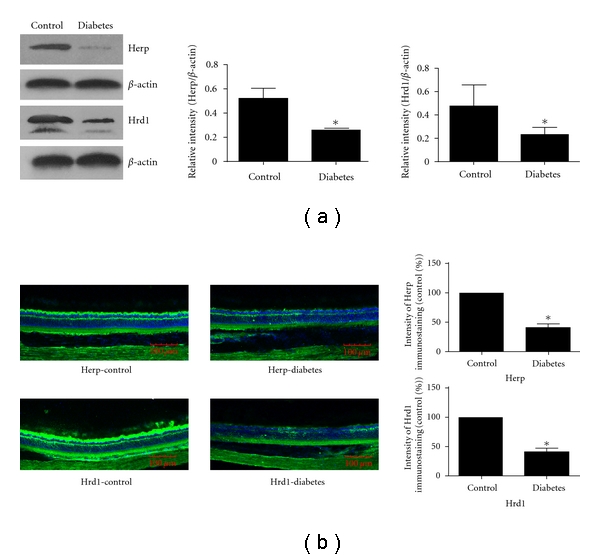
Western blot and immunofluorescence detected the expression of HERP and Hrd1 in the third month after the development of diabetes: (a) Western blot detection of the expression of HERP and Hrd1 in the third month. The expression of HERP and Hrd1in the diabetic group was less than that of the control group (*P* = 0.012 and *P* = 0.001, resp.). (b) immunofluorescence detection of the expression of HERP and HRD1 in the third month. The expression of HERP and Hrd1 in the diabetic group was less than that of the control group (*P* = 0.007 and *P* = 0.003, resp.).

We detected HERP and Hrd1 protein expression levels in the retinas of diabetic rats by Western blot and immunofluorescence in the first and the third months of diabetes development. The Western blot suggested that the HERP expression decreased significantly in the first month (*P* = 0.004) and third month (*P* = 0.012) compared with the nondiabetic control group. No significant change in the expression level of Hrd1 was observed in the first month (*P* = 0.338), while it decreased significantly in the third month compared with the control group (*P* = 0.001; Figures 2 and 3). 

The results of immunofluorescence were consistent with the Western blot. The protein level of HERP decreased significantly at both the first and third months (*P* = 0.008 and 0.007, resp.; Figures 2 and 3). There was no significant change in the expression of retinal HRD1 in the first month, while it decreased significantly in the third month (*P* = 0.572 and 0.003, resp. Figures 2 and 3).

### 3.3. Expression of HERP and HRD1 in RRCECs in the Presence of High-Glucose Concentration

**Figure 4:**
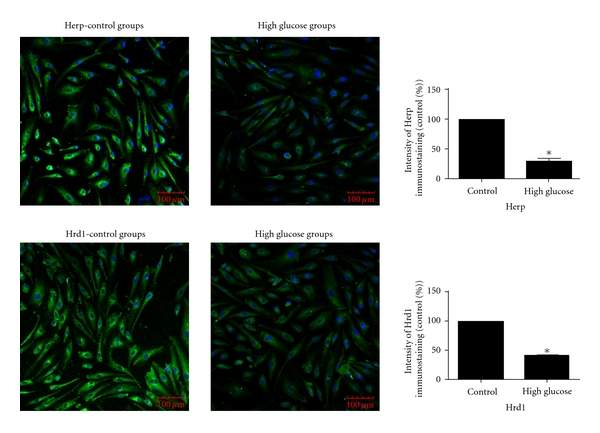
Immunofluorescence detection of the expression of HERP and HRD1 in RRCECs. The expression of HERP in the high glucose group was decreased compared to the control group, *P* = 0.013. The expression of HRD1 in the high glucose group was also decreased compared to the control group, *P* = 0.024.

The expression levels of HERP and HRD1 in RRCECs *in vitro* in the presence of high glucose concentration were decreased significantly compared to the control group (*P* = 0.013 and 0.024, resp.; Figure 4).

## 4. Discussion

The STZ-induced rat diabetes model is an established animal model for studying DR. Although we did not verify the development of DR in this study, our previous studies and the publication from another group have demonstrated that DR develops within one month of STZ-induced diabetes [13, 22, 23]. Our results indicate that of 89 ER stress genes, the expression of 12 genes in the retinas of diabetic rats was downregulated by the third month of diabetes development, and the expression of CCT4 increased within the first month. We did not observe any change in the expression of AFT4 or GFP78 at either time point in our study, which is consistent with our earlier results [13].

The expression of genes belonging to 8 different categories of ER stress factors was altered in the first month, while those of 10 categories were changed by the third month, suggesting that with increasing time more categories of ER stress factors were involved in the pathogenic process of DR. The expression of a number of related factors of the ERAD signaling pathways was downregulated, indicating that the ERAD signaling pathway may play an important role in DR. The ERAD system is an important pathway of protein degradation in the ER [25, 26] and plays important physiological roles. The ER is the location of protein synthesis, and secretion [27, 28] and has strict quality control mechanisms which allow secretion of correctly folded protein into the cytoplasm. The wrongly folded protein will be degraded through ERAD. ERAD therefore is a quality control system of the ER.

Recent studies found that HRD1 plays a central role in the ERAD-luminal pathway [29] and that HERP coordinates and regulates HRD1-mediated ubiquitylation [28], so we selected HRD1 and HERP from the ERAD pathway for further study. HERP expression was downregulated significantly in the retinas of diabetic rats in the first and third months. HERP is a membrane-bound, ubiquitin-like protein that is located in the ER. It forms a complex with ubiquitinated proteins and with the 26S proteasome [30–33]. HERP functions to degrade wrongly folded nonglycosylated proteins by forming a protein-enzyme complex with Derlin-1, HRD1, and p97 [34]. In our study, HRD1 expression in the retinas of diabetic rats remained unchanged in the first month, while it decreased in the third month. HRD1 is an E3 ubiquitin ligase and a key factor of ERAD [35–37]. ERAD has three pathways in yeast [38]: ERAD-L, ERAD-M, and ERAD-C. Both ERAD-L and ERAD-M are the key enzymes of HRD1. In the mammalian ERAD, HRD1 plays a very broad role in the ubiquitination process of abnormal proteins in the ER. The ubiquitin ligase HRD1 is mainly involved in the degradation of glycosylation proteins [39–41]. 

The decreased expression of HERP and HRD1 at both the mRNA and protein levels could lead to a decrease in function of ERAD's ability to remove wrongly folded proteins in the cell. Misfolded protein accumulation in the ER induces ER stress and activates signaling pathways, including PERK, ATF6, and IRE1 [15]. Persistent ER stress leads to cell death and induction of inflammation [42–45]. An inflammatory milieu is instrumental in breaking down the blood-retinal barrier in DR [46, 47]. 

In conclusion, we have shown by *in vivo* and *in vitro* experiments that an elevated concentration of glucose leads to downregulation of the ERAD signaling pathway. Such downregulation may result in local inflammation and DR.

## Figures and Tables

**Table 1:** Q-PCR arrays showed that the expression of the ER stress factor had significant differences in the first and the third month in diabetic rat retina: The ER stress factor of differential gene expression in the first month.

Symbol	Gene name	The average ratio of gene expression		*t*-test
		DR	control	*P* value
CCT4	*Cctd*	9.50E − 02	5.90*E* − 02	0.0134
DNAJB9	*Erdj4*	4.10E − 02	6.20*E* − 02	0.0125
DNAJC3	*P58IPK*	1.70E − 02	3.00*E* − 02	0.0173
Casp12	*Casp12*	3.20E − 04	1.30*E* − 03	0.002
ERP44	*Pdia10*	2.10E − 02	2.50*E* − 02	0.0337
GANAB	*GluII*	8.10E − 02	1.40*E* − 01	0.045
HERPUD1	*Herp*	1.50E − 01	2.60*E* − 01	0.0006
HSPA1L	*Hsp70-3*	6.30E − 04	1.00*E* − 03	0.0183
HSPA2	*Hspt70*	7.30E − 03	1.30*E* − 02	0.0183
MAPK8	*JNK*	1.10E − 02	2.00*E* − 02	0.0391
NUCB1	*NUC*	2.80E − 02	5.60*E* − 02	0.0289
OS9	*OS-9*	9.10E − 02	1.50*E* − 01	0.0272
SELS	*AD-015*	7.00E − 02	9.30*E* − 02	0.0486

**Table 2:** Q-PCR arrays showed that the expression of the ER stress factor had significant differences in the first and the third month in diabetic rat retina: The ER stress factor of differential gene expression in the third month.

Symbol	Gene name	The average ratio of gene expression		*t*-test
		DR	control	*P* value
ATF4	*CREB-2*	8.80E − 01	1.60E + 00	0.0178
DNAJB9	*Erdj4*	4.10E − 02	5.40E − 02	0.0106
ERO1L	*Ero1*	9.50E − 03	1.20E − 02	0.0492
TRB3	*Trib3*	5.80E − 03	6.10E − 02	0.0024
HERPUD1	*Herp*	7.20E − 02	2.80E − 01	0.0008
HTRA2	*PARK13*	2.20E − 02	4.00E − 02	0.0064
PPIA	*CYPA*	4.30E − 01	6.50E − 01	0.0238
SREBF1	*SREBP1*	2.10E − 02	2.80E − 02	0.0187
SYVN1/Hrd1	*HRD1*	7.90E − 02	1.20E − 01	0.0067
UFD1L	*UFD1*	1.60E − 01	8.00E − 02	0.0463
UGCGL1	*HUGT1*	9.80E − 03	2.10E − 02	0.0833
USP14	*TGT*	7.30E − 02	4.80E − 02	0.0405

**Table 3:** Q-PCR arrays showed that the expression of the ER stress factor had significant differences in the first and the third month in diabetic rat retina: the ER stress factor of significant differences belongs to different ER stress signaling pathways.

Signaling pathway	First month	Third month
Unfolded protein binding	*Cctd, ERdj4, Hspt70-3*	*ERdj4, OMI/PARK13, CYPA, HUGT1*
ER protein folding quality control	*GluII, PDIA10*	*HUGT1*
Regulation of cholesterol Metabolism	*—*	*SREBP1*
Regulation of translation	*—*	*—*
ERAD	*Herp, NUC, Os9, ADO15*	*Herp, OMI/PARK13, Hrd1*
Ubiquitination	*Herp*	*Herp, UfD1, TGT*
Transcription factors	*—*	*ATF4, SREBP1*
Protein folding	*Cctd, ERdj4,* *APG-1, PDIA10 *	*ERdj4, Ero1l, CYPA*
Protein disulfide isomerization	*PDIA10*	*SREBP1*
Heat shock proteins	*ERdj4, P58IPK, Hspt70-3*	*ERdj4*
Apoptosis	*JNK/JNK1, Casp12*	*OMI/PARK13, NIPK/Trib3*

**Table 4:** Q-PCR array gene table. We selected 89 ER stress-related factors, and other 6 genes as a quality control a total; of 96 genes were detected in Q-PCR arrays.

A01	Rn.107561	XM_341644	AMFR	Autocrine motility factor receptor	*AMFR*
A02	Rn.161941	NM_001108183	ARMET	Arginine rich, mutated in early-stage tumors	*ARMET*
A03	Rn.2423	NM_024403	ATF4	Activating transcription factor 4 (tax-responsive enhancer element B67)	*CREB-2/CREB2*
A04	Rn.222130	NM_001107196	ATF6	Activating transcription factor 6	*ATF6A*
A05	Rn.18179	NM_001002809	ATF6B	Activating transcription factor 6 beta	*CREB-RP/CREBL1*
A06	Rn.42932	NM_021702	ATXN3	Ataxin 3	*AT3/ATX3*
A07	Rn.10668	NM_017059	BAX	BCL2-associated X protein	*BCL2L4*
A08	Rn.974	NM_022399	CALR	Calreticulin	*CRT/RO*
A09	Rn.1762	NM_172008.2	CANX	Calnexin	*CNX/IP90*
A10	Rn.97889	NM_182814.2	CCT4	Chaperonin containing TCP1, subunit 4 (delta)	*CCT-DELTA/Cctd*
A11	Rn.62267	NM_001106603.1	CCT7	Chaperonin containing TCP1, subunit 7 (eta)	*CCT-ETA/Ccth*
A12	Rn.6479	NM_024125.4	CEBPB	CCAAT/enhancer binding protein (C/EBP), beta	*C/EBP-beta*
B01	Rn.104043	NM_001013092.1	CREB3	CAMP responsive element binding protein 3	*LUMAN/LZIP*
B02	Rn.20059	NM_001012115.1	CREB3L3	CAMP responsive element binding protein 3-like 3	*CREB-H/CREBH*
B03	Rn.11183	NM_001109986	DDIT3	DNA-damage-inducible transcript 3	*CEBPZ/CHOP*
B04	Rn.110990	NM_001014202.1	DERL1	Der1-like domain family, member 1	*DER-1/DER1*
B05	Rn.11209	NM_031627	CHOP	Rattus norvegicus nuclear receptor subfamily 1, group H, member 3	*LXRalpha/Nr1h3*
B06	Rn.40780	NM_001109541	DNAJB2	DnaJ (Hsp40) homolog, subfamily B, member 2	*HSJ1/HSPF3*
B07	Rn.29778	NM_012699	DNAJB9	DnaJ (Hsp40) homolog, subfamily B, member 9	*DKFZp564F1862/ERdj4*
B08	Rn.8642	NM_001106486	DNAJC10	DnaJ (Hsp40) homolog, subfamily C, member 10	*DKFZp434J1813/ERdj5*
B09	Rn.162234	NM_022232	DNAJC3	DnaJ (Hsp40) homolog, subfamily C, member 3	*HP58/P58*
B10	Rn.91398	NM_001013196	DNAJC4	DnaJ (Hsp40) homolog, subfamily C, member 4	*DANJC4/HSPF2*
B11	Rn.107459	NM_001033909	Elf2	E74-like factor 2	*Elf2*
B12	Rn.81078	NM_130422	Casp12	Caspase 12	*Casp12*
C01	Rn.198593	NM_001109339	eIF2A	Eukaryotic translation initiation factor 2A, 65 kDa	*CDA02/EIF-2A*
C02	Rn.24897	NM_031599	EIF2AK3	Eukaryotic translation initiation factor 2-alpha kinase 3	*DKFZp781H1925/HRI*
C03	Rn.19198	NM_001037208	CRELD2	cysteine-rich with EGF-like domains 2	*Creld2*
C04	Rn.218563	XM_344959.3	ERN2	Endoplasmic reticulum to nucleus signaling 2	*Ern2*
C05	Rn.64648	NM_138528	ERO1L	ERO1-like (*S. cerevisiae*)	*Ero1l*
C06	Rn.22325	NM_144755	TRB3	Tribbles homolog 3	*NIPK/Trib3*
C07	Rn.2459	NM_001008317	ERP44	Thioredoxin domain containing 4 (endoplasmic reticulum)	*PDIA10/TXNDC4*
C08	Rn.57325	NM_138917	FBXO6	F-box protein 6	*FBG2/FBS2*
C09	Rn.99241	NM_001106334	GANAB	Glucosidase, alpha; neutral AB	*G2AN/GluII*
C10	Rn.23744	NM_001145840	GANC	Glucosidase, alpha; neutral C	*MGC138256*
C11	Rn.4028	NM_053523	HERPUD1	Homocysteine-inducible, endoplasmic reticulum stress-inducible, ubiquitin-like domain member 1	*Sup*
C12	Rn.1950	NM_212504	HSPA1B	Heat shock 70 kDa protein 1B	*HSP70-1B/HSP70-2/Hsp72*
D01	Rn.187184	NM_212546	HSPA1L	heat shock protein 1-like	*Hsp70-3/MGC112562/MGC114222*
D02	Rn.211303	NM_021863	HSPA2	Heat shock protein 2	*Hspt70/Hst70/MGC93458*
D03	Rn.163092	NM_153629	HSPA4	Heat shock protein 4	*Hsp110/ Hsp70/irp94*
D04	Rn.144829	NM_001106428	HSPA4L	Heat shock protein 4-like	*APG-1; MGC187594; OSP94*
D05	Rn.11088	NM_013083	HSPA5	Heat shock 70 kDa protein 5 (glucose-regulated protein, 78 kDa)	*BIP/GRP78*
D06	Rn.37805	NM_001011901	HSPH1	Heat shock 105 kDa/110 kDa protein 1	*DKFZp686M05240/HSP105*
D07	Rn.107325	NM_001106599	HTRA2	HtrA serine peptidase 2	*OMI/PARK13*
D08	Rn.163330	NM_001107321	HTRA4	HtrA serine peptidase 4	*FLJ90724*
D09	Rn.772	NM_022392	INSIG1	Insulin-induced gene 1	*CL-6*
D10	Rn.16736	NM_178091	INSIG2	Insulin-induced gene 2	*MGC26273*
D11	Rn.9911	NM_012806	MAPK10	Mitogen-activated protein kinase 10	*JNK3/JNK3A*
D12	Rn.4090	XM_001056513	MAPK8	Mitogen-activated protein kinase 8	*JNK/JNK1*
E01	Rn.9910	NM_017322	MAPK9	Mitogen-activated protein kinase 9	*JNK-55/JNK2*
E02	Rn.2362	NM_053569	MBTPS1	Membrane-bound transcription factor peptidase, site 1	*PCSK8/S1P*
E03	Rn.212224	NM_001035007	MBTPS2	Membrane-bound transcription factor peptidase, site 2	*S2P*
E04	Rn.144645	NM_080577	NPLOC4	Nuclear protein localization 4 homolog (*S. cerevisiae*)	*NPL4*
E05	Rn.1492	NM_053463	NUCB1	Nucleobindin 1	*DKFZp686A15286/NUC*
E06	Rn.1579	NM_001007265	OS9	Osteosarcoma amplified 9, endoplasmic reticulum associated protein	*OS-9*
E07	Rn.11527	NM_017319	PDIA3	Protein disulfide isomerase family A, member 3	*ER60/ERp57*
E08	Rn.7627	NM_001109476	PFDN2	Prefoldin subunit 2	*PFD2*
E09	Rn.3401	NM_001106794	PFDN5	Prefoldin subunit 5	*MM-1/MM1*
E10	Rn.1463	NM_017101	PPIA	Peptidylprolyl isomerase A (cyclophilin A)	*CYPA/CYPH*
E11	Rn.2232	NM_133546	PPP1R15A	Protein phosphatase 1, regulatory (inhibitor) subunit 15A	*GADD34*
E12	Rn.104417	NM_001106806	PRKCSH	Protein kinase C substrate 80K-H	*AGE-R2/G19P1*
F01	Rn.209127	NM_001127545	RNF139	Ring finger protein 139	*HRCA1/RCA1*
F02	Rn.209127	NM_006913	RNF5	Ring finger protein 5	*RING5/RMA1*
F03	Rn.4224	NM_013067	RPN1	Ribophorin I	*DKFZp686B16177/OST1*
F04	Rn.99548	NM_001100966	SCAP	SREBF chaperone	*KIAA0199*
F05	Rn.98327	NM_001034129	SEC62	SEC62 homolog (*S. cerevisiae*)	*Dtrp1/HTP1*
F06	Rn.24233	NM_001107637	SEC63	SEC63 homolog (*S. cerevisiae*)	*ERdj2/PRO2507*
F07	Rn.20802	NM_177933	SEL1L	Sel-1 suppressor of lin-12-like (*C. elegans*)	*IBD2/PRO1063*
F08	Rn.4197	NM_173120	SELS	Selenoprotein S	*AD-015/ADO15*
F09	Rn.2119	NM_030835	SERP1	Stress-associated endoplasmic reticulum protein 1	*RAMP4*
F10	Rn.103851	NM_199376	SIL1	SIL1 homolog, endoplasmic reticulum chaperone (*S. cerevisiae*)	*BAP/MSS*
F11	Rn.221929	XM_001075680	SREBF1	Sterol regulatory element binding transcription factor 1	*SREBP-1c/SREBP1*
F12	Rn.41063	NM_001033694	SREBF2	Sterol regulatory element binding transcription factor 2	*SREBP2/bHLHd2*
G01	Rn.162486	NM_001100739	SYVN1	Synovial apoptosis inhibitor 1, synoviolin	*HRD1*
G02	Rn.7102	NM_012670	TCP1	T-complex 1	*CCT-alpha/CCT1*
G03	Rn.20041	NM_153303	TOR1A	Torsin family 1, member A (torsin A)	*DQ2/DYT1*
G04	Rn.139603	NM_001106380	UBE2G2	Ubiquitin-conjugating enzyme E2G 2 (UBC7 homolog, yeast)	*UBC7*
G05	Rn.106299	NM_001007655	UBE2J2	Ubiquitin-conjugating enzyme E2, J2 (UBC6 homolog, yeast)	*NCUBE2/PRO2121*
G06	Rn.2022	NM_001012025	UBXN4	UBX domain protein 4	*UBXD2/UBXDC1*
G07	Rn.11946	NM_053418	UFD1L	Ubiquitin fusion degradation 1-like (yeast)	*UFD1*
G08	Rn.162227	NM_133596	UGCGL1	UDP-glucose ceramide glucosyltransferase-like 1	*HUGT1*
G09	Rn.107678	NM_019381	BI-1	Transmembrane BAX inhibitor motif containing 6	*Tmbim6*
G10	Rn.11790	NM_001008301	USP14	Ubiquitin-specific peptidase 14 (tRNA-guanine transglycosylase)	*TGT*
G11	Rn.98891	NM_053864	VCP	Valosin-containing protein	*IBMPFD/TERA*
G12	Rn.101044	NM_001004210	XBP1	X-box binding protein 1	*TREB5/XBP2*
H01	Rn.973	NM_001007604	Rplp1	Ribosomal protein, large, P1	*MGC72935*
H02	Rn.47	NM_012583	Hprt	Hypoxanthine guanine phosphoribosyl transferase	*Hgprtase/Hprt1*
H03	Rn.92211	NM_173340	Rpl13a	Ribosomal protein L13A	*Rpl13a*
H04	Rn.107896	NM_017025	Ldha	Lactate dehydrogenase A	*Ldh1*
H05	Rn.94978	NM_031144	Actb	Actin, beta	*Actx*
H06	N/A	U26919	RGDC	Rat genomic DNA contamination	*RGDC*
H07	N/A	SA_00104	RTC	Reverse Transcription Control	*RTC*
H08	N/A	SA_00104	RTC	Reverse transcription control	*RTC*
H09	N/A	SA_00104	RTC	Reverse transcription control	*RTC*
H10	N/A	SA_00103	PPC	Positive PCR control	*PPC*
H11	N/A	SA_00103	PPC	Positive PCR control	*PPC*
H12	N/A	SA_00103	PPC	Positive PCR control	*PPC*
